# First-line pemetrexed and carboplatin plus anlotinib for epidermal growth factor receptor wild-type and anaplastic lymphoma kinase-negative lung adenocarcinoma with brain metastasis: A case report and review of the literature

**DOI:** 10.1097/MD.0000000000022128

**Published:** 2020-09-04

**Authors:** Chu Zhang, Feng-Wei Kong, Wen-Bin Wu, Miao Zhang, Guang-Mao Yu, Xiang Wang, Yuan-Yuan Liu

**Affiliations:** a Department of Thoracic Surgery, Shaoxing People's Hospital (Shaoxing Hospital of Zhejiang University), Shaoxing; b Department of General Surgery, Xuzhou Infectious Disease Hospital; c Department of Respirology and Critical Care Medicine, Xuzhou Central Hospital, Xuzhou, China.

**Keywords:** anlotinib (AL3818), lung cancer, pemetrexed, targeted therapy, tyrosine kinase inhibitor, vascular endothelial growth factor receptor

## Abstract

**Rationale:**

Brain metastasis (BM) is a serious complication in non-small cell lung cancer (NSCLC) patients. Pemetrexed is one of the preferred agents in nonsquamous NSCLC with BM; however, the traditional chemotherapy demonstrated limited efficacy partly due to drug resistance and the blood-brain barrier.

**Patient concerns:**

A 52-year-old male non-smoker was admitted for irritating cough, chest distress, and back pain.

**Diagnoses:**

Epidermal growth factor receptor wild-type, anaplastic lymphoma kinase-negative primary lung adenocarcinoma with an asymptomatic solitary BM (cTxNxM1b, IVA).

**Interventions:**

Pemetrexed (500 mg/m^2^ of body surface area) and carboplatin (area under the curve of 5) were firstly administered every 3 weeks for 3 cycles, followed by pemetrexed/carboplatin plus anlotinib (12 mg daily; 2 weeks on and 1 week off) for another 3 cycles. Then maintenance anlotinib monotherapy was continued for a year, without unacceptable adverse events.

**Outcomes:**

The BM was slightly enlarged after 3 cycles of pemetrexed/carboplatin; however, a complete remission was achieved after the combination therapy. His intracranial progression-free survival was more than 2 years.

**Lessons:**

Pemetrexed/carboplatin plus anlotinib could be considered for the treatment of epidermal growth factor receptor wild-type, anaplastic lymphoma kinase-negative lung adenocarcinoma with BM. Further well-designed trials are warranted to verify this occasional finding.

## 1 Introduction

Anlotinib (AL3818) is a tyrosine kinase inhibitor (TKI) that targets vascular endothelial growth factor receptor, fibroblast growth factor receptor, platelet-derived growth factor receptors, and c-kit. Anlotinib has been approved in China for locally advanced or metastatic non-small cell lung cancer (NSCLC) patients who have undergone tumor progression or recurrence after ≥2 lines of systemic chemotherapy,^[1]^ which is based on a significant improved overall survival (OS) with anlotinib versus the placebo. It is reported that the major toxicities of anlotinib include hypertension (67.4%), hand-foot syndrome (43.9%), hemoptysis (14.0%), thyroid stimulating hormone elevation (46.6%), and corrected QT interval prolongation (26.2%).^[2]^ At the dose of 12 mg once daily at the 2-week on and 1-week off schedule, anlotinib displays manageable toxicity, long circulation, and broad-spectrum antitumor efficacy.^[3]^ In detail, the plasma concentrations of anlotinib reached its maximum on day 14 and decreased subsequently until the next cycle of treatment. Although it improves the progression-free survival (PFS) and OS of the patients with advanced NSCLC, anlotinib has a significantly lower incidence of grade 3 or higher side effects compared to sunitinib.^[4]^

However, for epidermal growth factor receptor (EGFR) wild-type NSCLC patients, the therapeutic options for brain metastasis (BM) are limited. Pemetrexed (combined with cisplatin or carboplatin) is the first-line agent for lung adenocarcinoma according to the National Comprehensive Cancer Network guideline for NSCLC, Version 1.2020^[5]^; nevertheless, the efficacy of this traditional chemotherapy regimen in EGFR-negative, nonsquamous NSCLC patients with BM is somewhat uncertain.

To the best of our knowledge, the evidence concerning the efficacy of first-line pemetrexed plus anlotinib for BM from nonsquamous NSCLC is still lacking. Herein we presented a lung adenocarcinoma patient who demonstrated a complete remission of a solitary BM for 2 years after pemetrexed/carboplatin plus anlotinib. Furthermore, the relevant reports and the registered trials in terms of the TKI-based treatments for BM from lung cancer were briefly reviewed.

## 2 Case presentation

**Figure 1:**
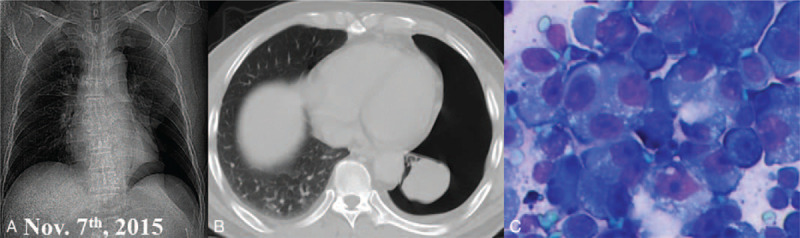
The chest images and cytology of pleural effusion. (A) The chest x-ray radiography showed left-sided pleural effusion and atelectasis of the left lower lobe (November 2015); (B) The computed tomography indicated the atelectasis of the left lower lobe after closed thoracic drainage; (C) The cytology of the drained effusion revealed malignant cells (hematoxylin-eosin staining, ×400).

The clinical data were treated anonymously for privacy concern. A 52-year-old male nonsmoker was admitted due to irritating cough, chest distress, and back pain in November 2015. The chest x-ray indicated left-sided pleural effusion and atelectasis of the left lower pulmonary lobe (Fig. 1A). Laboratory tests indicated mainly normal serum neuron-specific enolase, carcinoembryonic antigen, carbohydrate antigen 724/125, alkaline phosphatase, cytokeratin-19 fragment, aspartate aminotransferase, alanine aminotransferase, gamma-glutamyl transpeptidase, lactic dehydrogenase, and albumin.

**Figure 2:**
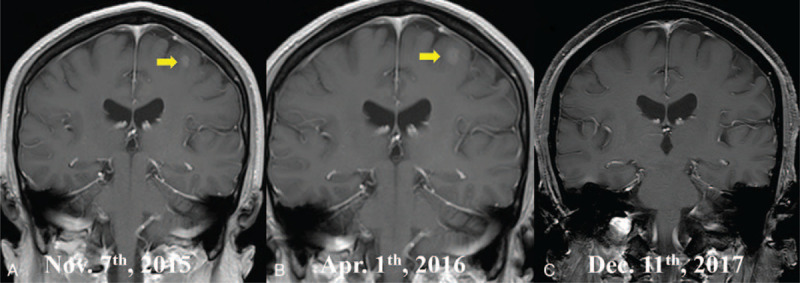
The brain magnetic resonance imaging. (A) A solitary BM was indicated before the therapy (labeled by the yellow arrow); (B) The intracranial lesion maintained stable after 3 cycles of pemetrexed/carboplatin (labeled by the yellow arrow); (C) A complete remission of the BM was demonstrated 2 yr after the combination treatment using pemetrexed and anlotinib for 3 cycles and maintained anlotinib monotherapy for 1 yr. BM = brain metastasis.

The patient was initially diagnosed as spontaneous hydropneumothorax empirically. Further contrast-enhanced computed tomography after chest tube drainage showed atelectasis (Fig. 1B). In addition, malignant tumor cells were detected in the pleural effusion (Fig. 1C), which supported the pathological diagnosis of primary lung adenocarcinoma. Moreover, the cranial magnetic resonance imaging revealed a solitary BM in the left cerebrum (Fig. 2A); whereas the whole-body emission computed tomography excluded other metastases. However, a definite diagnosis was not obtained, because a thoracoscopic biopsy was not performed to avoid unnecessary injury and to diminish the risk of iatrogenic tumor dissemination. Based on these findings, this case was staged as cTxNxM1b, IV A according to the 8th edition of tumor, node, and metastasis staging system for lung cancer.^[6]^ Liquid biopsy showed wild-type EGFR, human epidermal growth factor receptor 2 and vascular endothelial growth factor, followed by negative echinoderm microtubule-associated protein-like 4-anaplastic lymphoma kinase (ALK) fusion gene.

After a multidisciplinary evaluation, first-line systemic anti-cancer treatment, instead of surgery for the cranial oligometastasis, was scheduled. Informed consent was obtained from the patient before treatment. The efficacy was evaluated according to the Response Evaluation Criteria in Solid Tumors version 1.1; meanwhile, the adverse events were recorded and staged in line with the National Cancer Institute Common Terminology Criteria for Adverse Events version 4.0.

Initially, the patient was given first-line pemetrexed (500 mg/m^2^ of body surface area) and carboplatin (Area under curve (AUC) = 5) every 21 days following the prophylactic folic acid and vitamin B12 for 3 cycles. There was no newly-emerged lung or pleural lesions, which might revealed the efficacy of intravenous pemetrexed/carboplatin in lung adenocarcinoma. However, the solitary BM showed stable disease after the first 3 cycles of chemotherapy (Fig. 2B).

After another multidisciplinary consultation, pemetrexed/carboplatin plus oral anlotinib (12 mg once daily at 2-week on and 1-week off schedule) was administered. Reimaging of the brain after another 3 cycles of combination therapy demonstrated an impressive complete remission of the BM (Fig. 2C). Subsequently, maintenance anlotinib monotherapy, instead of pemetrexed, was continued for 1 year. During the follow up, there was no detectable pleural effusion, newly-onset lung lesion, or distant metastasis. The adverse events were mainly well tolerated. Grade 2/3 hypertension and hand-foot syndrome were controlled effectively. No grade 4 toxicities were observed in this case. The patient obtained an intracranial PFS of more than 2 years till February 2018; however, he was lost to follow up thereafter.

## 3 Discussion

BM from lung cancer is associated with poor survival of the patients. The incidence of BM has continued to rise, as most patients develop resistance to targeted agents.^[7]^ About 10% of NSCLC patients have BM at diagnosis while 25% to 40% of them develop BM; however, the conventional chemotherapy does not cross the blood-brain barrier.^[8]^ To date, the established management approaches for BM include stereotactic radiosurgery, fractionated radiation therapy, and surgical resection^[9]^; nevertheless, the optimal regimen for achieving long-term control of BM is yet to be elucidated. In the present case, anlotinib plus pemetrexed/carboplatin demonstrated enduring efficacy in lung adenocarcinoma with solitary BM.

Pemetrexed disodium is effective in various solid tumors. However, the distribution of pemetrexed into the central nervous system is truly limited, probably due to an efflux clearance process.^[10]^ Another research revealed that pemetrexed could distributed from the plasma to the cerebrospinal fluid (CSF) within 1 to 4 hours, but the concentrations of this agent in the CSF was less than 5% of that in the plasma; therefore, the limited anti-tumor activity of intravenous pemetrexed might partly due to its low concentration in the CSF.^[11]^ Novel therapeutic agents must cross the blood vessel wall to reach cancer cells in adequate quantities and overcome the acquired drug resistance. The studies of BM could uncover new therapeutic targets and identify treatment approaches.^[12]^ A deep understanding of the blood-brain barrier and blood-tumor barrier would enable personalized management for primary brain malignancies and BMs,^[13]^ because the utilization of small-molecules drugs or proteins for the treatment of central nervous system tumors is significantly restricted by the blood-brain barrier.

A retrospective analysis of NSCLC patients with BM and EGFR mutation showed that the concurrent EGFR-TKI and whole-brain radiotherapy improved the oncological benefits without additional adverse events.^[14]^ Nonetheless, emerging data revealed that the whole-brain radiotherapy is associated with high incidences of neurotoxicity; therefore, despite the improvements in targeted therapy and immunotherapy, new agents that target the genetic mutations enriched in BM are still urgently needed.^[15]^ Besides the intracranial activity of immunotherapy, there is growing evidence indicating that TKIs used in patients with identified targetable genetic mutations or rearrangements could be effective in the central nervous system. It is reported that the number of BM does not impact the oncological prognosis in the EGFR/ALK mutated NSCLC patients; in addition, the number of BM independently affect the survival in driver gene wild-type BM patients.^[16]^ The ALK inhibitors including alectinib, ceritinib, brigatinib, lorlatinib have been designed to cross the blood-brain barrier more efficiently than crizotinib and achieve higher concentration in the CSF.^[17]^ A pooled analysis of 2 trials confirmed the safety and efficacy of second-line bevacizumab and pemetrexed in NSCLC patients with BM.^[18]^

We searched PubMed, Web of Science, Scopus, Embase, Europe PMC, Cochrane Library, and Google Scholar for similar studies regarding pemetrexed and targeted therapy for advanced NSCLC with BM up to February 2020. Keywords and MeSH terms in title or abstract including “TKI” or “pemetrexed” or “targeted therapy” and “pulmonary” or “lung” and “cancer” and “brain metastasis” or “cranial metastasis” were used. No restriction was made regarding the publication languages. Finally a total of 17 reports involving 369 patients were summarized and listed in Table 1, which demonstrated the efficacy of combination therapeutic regimen in lung cancer with BM. Specifically, Yu et al reported that first-line pemetrexed-based chemotherapy provided a median OS and intracranial PFS of 21.0 months and 9.5 months respectively in 138 NSCLC patients with BM.^[34]^ Furthermore, a retrospective cohort study showed that the overall cumulative incidence of BM was significantly higher in the targeted therapy group than those in the cytotoxic chemotherapy group, whereas the younger age, female, and first-line targeted therapy were significant risk factors of subsequent BM.^[35]^

**Table 1:**
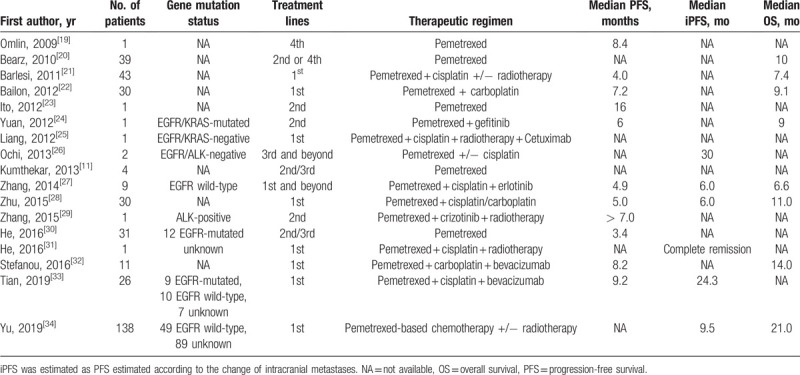
Previous reports of pemetrexed and targeted therapy for advanced NSCLC with brain metastases.

However, a pooled analysis or meta-analysis was not applicable because most of the survival data of the patients were not available from these articles. Considering the generally low quality of evidence from the retrieved studies, more trials are warranted. The registered trials regarding anlotinib (AL3818) or pemetrexed for the treatment of lung cancer with BM was listed in Table 2 and Table 3, respectively. Accordingly, an updated guideline or consensus recommendation using targeted therapy plus pemetrexed for the treatment of lung cancer with BM might be provided based on the ongoing evidence.

**Table 2:**
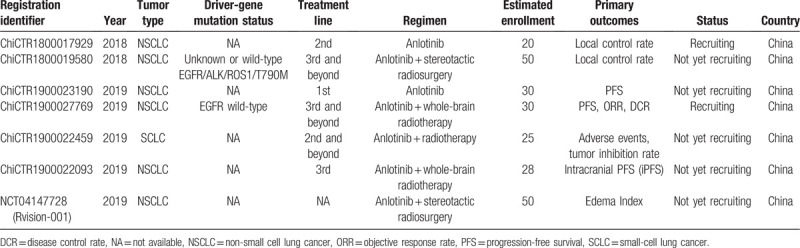
The registered trials regarding anlotinib (AL3818) in the treatment of lung cancer with brain metastases.

**Table 3:**
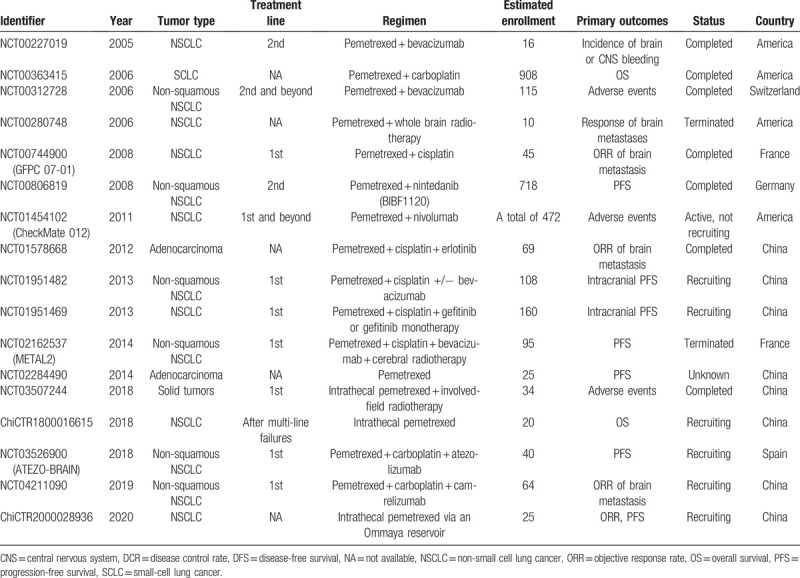
The registered trials of pemetrexed for lung cancer patients with brain metastases.

## 4 Conclusions

Pemetrexed/carboplatin plus anlotinib could be considered for patients with EGFR wild-type, ALK-negative lung adenocarcinoma, and BM. Further well-designed trials are warranted.

## Author contributions

**Conceptualization:** Chu Zhang, Feng-Wei Kong, Xiang Wang.

**Data curation:** Guang-Mao Yu.

**Funding acquisition:** Chu Zhang, Miao Zhang.

**Methodology:** Yuan-Yuan Liu.

**Resources:** Xiang Wang, Wen-Bin Wu.

**Writing – original draft:** Feng-Wei Kong, Wen-Bin Wu, Xiang Wang.

**Writing – review & editing:** Chu Zhang, Feng-Wei Kong, Yuan-Yuan Liu.
